# Pediatric Familial Cerebral Cavernous Malformation Associated With a Novel *KRIT1* Initiation‐Region Frameshift Variant

**DOI:** 10.1002/mgg3.70304

**Published:** 2026-09-17

**Authors:** Özlem Yayıcı Köken, Mehpare Sarı Yanartaş, Hande Aygün, Ahmet Cevdet Ceylan, Kamil Karaali, Mehmet Saim Kazan

**Affiliations:** ^1^ Division of Pediatric Neurology, Department of Pediatrics Akdeniz University Faculty of Medicine Antalya Turkey; ^2^ Division of Pediatric Neurology, Department of Pediatrics Prof. Dr. Süleyman Yalçın City Hospital İstanbul Turkey; ^3^ Department of Medical Genetics Yıldırım Beyazıt University Faculty of Medicine, Ankara Bilkent City Hospital Ankara Turkey; ^4^ Department of Radiology Akdeniz University Faculty of Medicine Antalya Turkey; ^5^ Department of Neurosurgery Akdeniz University Faculty of Medicine Antalya Turkey

**Keywords:** cascade screening, cerebral cavernous malformation, familial cerebral cavernous malformation, *KRIT1*, whole‐exome sequencing

## Abstract

**Background:**

Familial cerebral cavernous malformation (CCM) is an autosomal dominant vascular disorder with age‐dependent penetrance and marked intrafamilial variability. *KRIT1* loss‐of‐function variants are the most common genetic cause, but pediatric genotype–phenotype correlations remain limited.

**Methods:**

Clinical, radiological, histopathological, and molecular findings of a pediatric family with suspected familial CCM were reviewed. Brain and spinal MRI, calvarial histopathology, whole‐exome sequencing, segregation analysis, and targeted literature review were performed.

**Results:**

The index patient was a 14‐year‐old boy presenting with focal seizure and a progressively enlarging right parietal calvarial mass. MRI revealed multiple cerebral and cerebellar CCMs with a cervical intramedullary cavernous malformation. The calvarial lesion was excised and confirmed as hemangioma. Whole‐exome sequencing identified a previously unreported heterozygous *KRIT1* initiation‐region duplication, NM_004912.4: c.2dup, predicted to result in p.(Met1IlefsTer31) with loss of all major functional domains. The same variant was detected in the clinically asymptomatic sibling with MRI‐confirmed multiple CCMs, whereas the mother was negative. The father had died from intracerebral hemorrhage, but genetic testing was unavailable.

**Conclusion:**

This report expands the *KRIT1* mutational spectrum and illustrates the wide clinical range of familial CCM, from asymptomatic radiological disease to epilepsy and fatal hemorrhage within one family. These findings support early molecular diagnosis, cascade screening, and susceptibility‐sensitive MRI surveillance.

## Graphical abstract

A novel *KRIT1* initiation‐region frameshift variant, NM_004912.4.2dup [p.(Met1IlefsTer31)], was identified in a pediatric family with familial cerebral cavernous malformation. Marked intrafamilial variability supports early molecular diagnosis, cascade family screening, and susceptibility‐sensitive MRI surveillance.

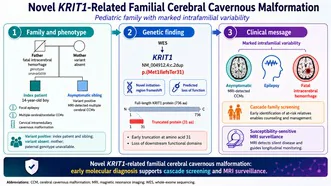

## 1 Introduction

Cerebral cavernous malformations (CCMs) are vascular lesions characterized by clusters of dilated capillary channels that may occur sporadically or as part of a familial disorder. Familial CCM is inherited in an autosomal dominant manner and is primarily associated with pathogenic variants in *KRIT1* (*CCM1*), *CCM2*, and *PDCD10* (*CCM3*), with *KRIT1* accounting for the majority of genetically confirmed familial CCM (Yang et al. 2016; Lucas et al. 2003; Baranoski et al. 2016; Marchuk et al. 2003). These genes play critical roles in maintaining endothelial cell integrity and vascular stability, and pathogenic loss‐of‐function variants predispose affected individuals to the development of multiple CCM lesions (Baranoski et al. 2016; Marchuk et al. 2003; Zhang et al. 2020; Chen et al. 2023). In particular, *KRIT1* loss‐of‐function variants are a well‐established cause of familial CCM, and variants affecting the translation initiation region are predicted to disrupt normal protein production and function (Pileggi et al. 2010).

The clinical presentation of CCM is highly variable, ranging from asymptomatic disease to seizures, focal neurological deficits, headaches, and intracranial hemorrhage (Pileggi et al. 2010; Merello et al. 2016; Ricci, Cerase, et al. 2021; Wang et al. 2017). In pediatric populations, seizures are among the most common manifestations of supratentorial lesions, whereas brainstem lesions are more frequently associated with hemorrhagic presentations and neurological deficits (Pileggi et al. 2010; Russo et al. 2017; Buckingham et al. 1989; Akers et al. 2017). Spinal cavernous malformations are less common but may occur in familial disease.

Magnetic resonance imaging (MRI), particularly susceptibility‐sensitive sequences such as gradient‐echo and susceptibility‐weighted imaging (SWI), has substantially improved the detection of CCMs and facilitated recognition of familial forms characterized by multifocal lesions (Riolo et al. 2021; Lanfranconi et al. 2021; Ricci, Riolo, and Battistini 2021). Molecular genetic testing further enhances diagnostic accuracy by enabling identification of affected relatives and supporting genetic counseling (Marchuk et al. 2003; Ricci, Riolo, and Battistini 2021; Haasdijk et al. 2012). Management depends on lesion location, symptom burden, and hemorrhagic risk, ranging from conservative clinical and radiological follow‐up to surgical intervention for medically refractory epilepsy, recurrent hemorrhage, or progressive neurological deficits (Akers et al. 2017; Horne et al. 2016; Boulday and Tournier‐Lasserve 2020; Alexiou and Prodromou 2011; Mottolese et al. 2001).

Familial CCM exhibits age‐dependent penetrance and variable clinical expression, even among affected members of the same family. Continued reporting of genetically characterized families is important for refining genotype–phenotype correlations, expanding the mutational spectrum of CCM‐associated genes, and improving clinical management. Here, we describe a pediatric family with CCM associated with a *KRIT1* c.2dup variant affecting the translation initiation region. The family demonstrated marked intrafamilial phenotypic variability, ranging from asymptomatic radiological disease to epilepsy and fatal intracerebral hemorrhage in a first‐degree relative.

## 2 Materials and Methods

### 2.1 Editorial Policies and Ethical Considerations

Ethics committee approval was not required for this single‐family clinical report according to institutional policy; however, all procedures were conducted in accordance with the Declaration of Helsinki. Written informed consent was obtained prior to inclusion from the parents/legal guardians of the index patient and from participating family members for the analysis and publication of clinical, radiological, histopathological, and genetic data, including clinical images and family‐based genetic findings. All personal identifiers were removed, and family members were described using non‐identifying clinical designations to preserve confidentiality.

Clinical, radiological, histopathological, and molecular findings of the index patient and affected family members were reviewed. Brain MRI, histopathological examination of the calvarial lesion, whole‐exome sequencing, and cascade genetic testing were performed. A structured literature search was performed in PubMed, ClinVar, and HGMD databases using the terms “*KRIT1*,” “*CCM1*,” “cerebral cavernous malformation,” and “translation initiation variant.”

### 2.2 Genetic Testing

Whole‐exome sequencing (WES) was performed using the GeneMind Whole Exome Sequencing Kit (GeneMind Biosciences, Shenzhen, China) on the Genanalyser platform. Sequence reads were aligned to the GRCh38/hg38 human reference genome using the Burrows–Wheeler Aligner (BWA‐MEM). The mean sequencing depth was 125.83× in the proband, 131.74× in the clinically asymptomatic sibling, and 111.89× in the mother. In the proband, target‐region coverage was 99.80% at ≥ 1×, 99.09% at ≥ 10×, and 97.74% at ≥ 20×. In the clinically asymptomatic sibling, target‐region coverage was 99.80% at ≥ 1×, 99.18% at ≥ 10×, and 98.09% at ≥ 20×. In the mother, target‐region coverage was 99.71% at ≥ 1×, 98.78% at ≥ 10×, and 97.22% at ≥ 20×. Variant interpretation was performed according to the American College of Medical Genetics and Genomics/Association for Molecular Pathology (ACMG/AMP) guidelines. Familial segregation analysis was performed using high‐quality WES data obtained from the available family members. The identified *KRIT1* variant demonstrated complete segregation with the disease phenotype, being present in both affected siblings and absent in the clinically unaffected mother. Given the high sequencing quality (mean coverage > 110× and > 97% target‐region coverage at ≥ 20×) and the concordant segregation findings, additional Sanger sequencing confirmation was not performed. *KRIT1* variant nomenclature was based on the GenBank reference transcript NM_004912.4 and followed current Human Genome Variation Society (HGVS) recommendations.

## 3 Results

### 3.1 Clinical Presentation of the Index Patient

A 14‐year‐old boy was referred to our pediatric neurology clinic after a focal epileptic seizure associated with left arm numbness. Review of prior medical records showed that 5 months earlier, he had been evaluated by the neurosurgery department because of a painful, progressively enlarging right parietal scalp mass. According to the family, the lesion had been palpable for approximately 2 years and had enlarged more rapidly during the preceding year before surgical removal. Physical examination at that time revealed a firm, painful subcutaneous mass measuring approximately 5 cm in the right parietal region, with a broad‐based attachment to the dura. No additional neurological deficits were documented. Family history was notable for the father's sudden death at 41 years of age due to an intracerebral hemorrhage.

**FIGURE 1:**
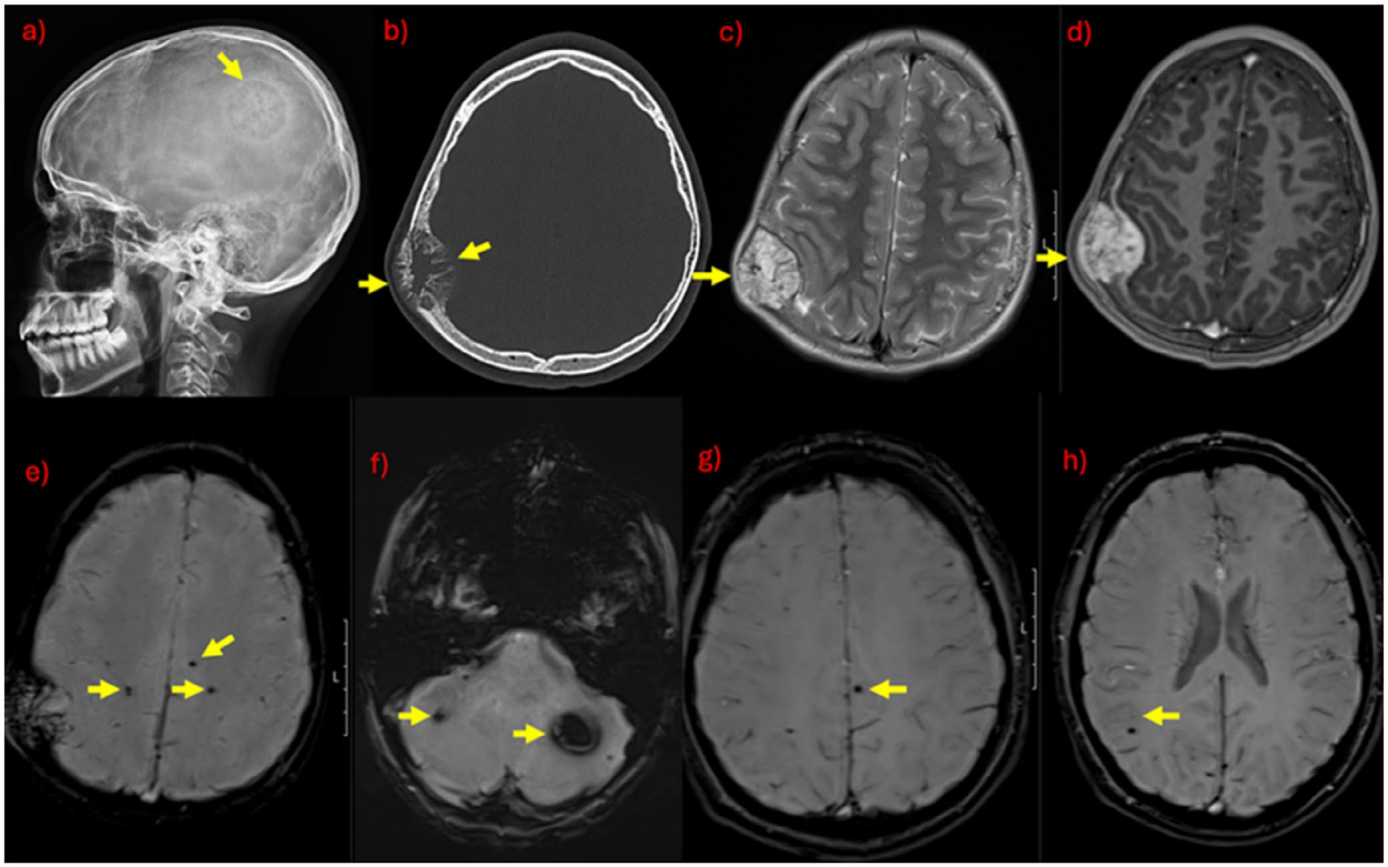
Multimodal imaging findings in the index patient and the clinically asymptomatic sibling with familial cerebral cavernous malformations (CCMs). (a–f) Imaging of the index patient. (a) Lateral skull radiograph demonstrating a well‐circumscribed lesion in the right parietal calvarium with radiating trabecular striations producing a characteristic “spoke‐wheel” pattern within the diploic space, typical of calvarial intraosseous hemangioma (arrows). (b) Axial bone‐window CT image showing an expansile lesion of the right parietal bone with radiating trabecular thickening and expansion of the diploe, consistent with calvarial intraosseous hemangioma (arrows). (c) Axial T2‐weighted MRI demonstrating a well‐defined lesion within the right parietal calvarium with expansion of the diploic space and heterogeneous high T2 signal with internal trabecular architecture (arrows). (d) Axial T1‐weighted post‐contrast MRI showing strong enhancement of the calvarial lesion (arrows). (e) Axial susceptibility‐weighted imaging (SWI) demonstrating multiple small punctate hypointense foci within the cerebral parenchyma, consistent with CCMs (arrows). (f) Axial SWI MRI demonstrating bilateral cerebellar hypointense lesions consistent with CCMs (arrows). (g–h) Imaging of the asymptomatic sibling carrying the same *KRIT1* variant. Axial SWI MRI demonstrating multiple punctate hypointense foci within the cerebral parenchyma consistent with multiple CCMs (arrows).

**FIGURE 2:**
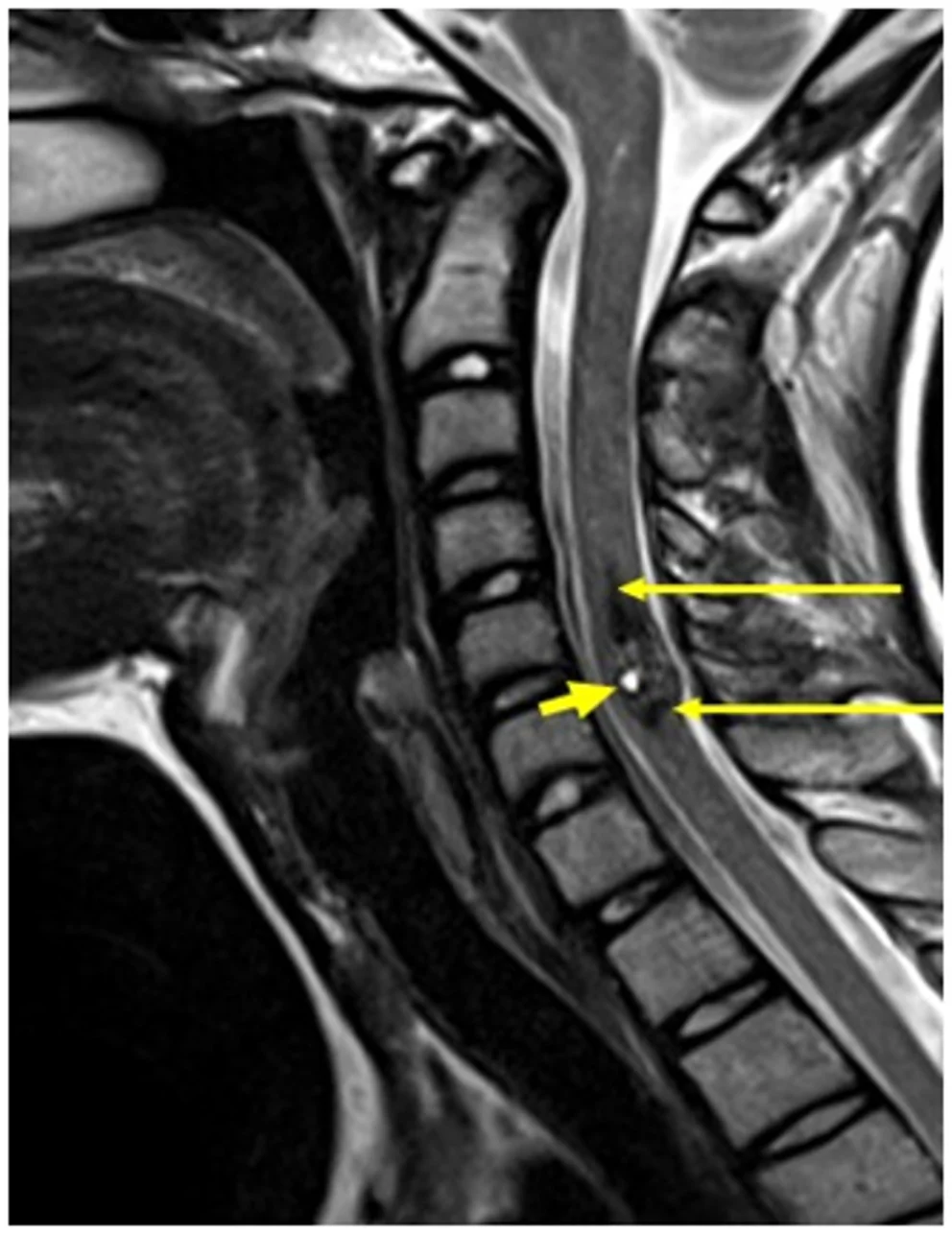
Sagittal T2‐weighted MRI of the cervical spine demonstrating an intramedullary lesion with a central hyperintense core (arrowhead) surrounded by a hypointense hemosiderin rim (arrows), consistent with a spinal cord cavernous malformation.

Imaging obtained during the earlier neurosurgical evaluation is shown in Figure 1a–f. Lateral skull radiography and bone‐window cranial CT demonstrated a well‐circumscribed expansile lesion of the right parietal calvarium with radiating trabecular striations and diploic expansion, consistent with calvarial intraosseous hemangioma (Figure 1a,b). Cranial MRI confirmed a well‐defined right parietal calvarial lesion with diploic expansion, heterogeneous high T2 signal, and strong post‐contrast enhancement (Figure 1c,d). Susceptibility‐weighted imaging also revealed multiple small punctate hypointense foci within the cerebral parenchyma and bilateral cerebellar hypointense lesions, consistent with CCMs (Figure 1e,f). Based on imaging characteristics and anatomical location, the calvarial lesion was interpreted as an intraosseous hemangioma unrelated to the intracranial cavernous malformations. Histopathological examination of the resected calvarial lesion, obtained from an 8 × 7.4 × 3 cm bone excision biopsy specimen, was compatible with hemangioma. In addition to the cerebral lesions, spinal MRI revealed a cervical intramedullary cavernous malformation (Figure 2), further supporting the diagnosis of a multifocal familial cavernous malformation syndrome.

5 months later, he presented with a focal epileptic seizure characterized by left arm numbness. EEG showed focal epileptiform abnormalities in the right temporoparietal region consistent with seizure semiology. Levetiracetam was initiated, and he has remained seizure‐free for 6 months. Apart from headache, he reported no additional neurological complaints. He described throbbing headaches occurring 2–3 times per week, usually in the afternoon, with more severe weekly episodes lasting 3–4 h and responding to paracetamol. Further evaluation revealed no cutaneous or retinal hemangioma and no focal motor neurological deficit. Current neurological examination is normal.

The intracranial CCMs were asymptomatic, and the patient was placed on radiological follow‐up. Given the family history of fatal intracerebral hemorrhage and the presence of multiple cerebral cavernous malformations in the proband, familial CCM was suspected and WES was performed. This finding also prompted genetic and radiological evaluation of available family members. Genetic analysis identified the same heterozygous *KRIT1* c.2dup variant in the proband's older sibling. Although clinically asymptomatic and without a history of seizures, headaches, or focal neurological deficits, cranial MRI revealed multiple cerebral cavernous malformations, indicating radiologically affected familial CCM despite the absence of neurological symptoms (Figure 1g,h). In contrast to the proband, spinal MRI showed no evidence of spinal cord involvement. The clinical, radiological, and genetic characteristics of the available family members are summarized in Table 1.

**TABLE 1:** Clinical, radiological, and genetic characteristics of the available family members.

Individual	Age/Sex	*KRIT1* Variant	Clinical Features	Neuroimaging findings	Outcome/current status
Index patient (proband)	14/M	Heterozygous NM_004912.4: c.2dup, p.(Met1IlefsTer31)	Focal epileptic seizure with left arm numbness; recurrent headache; painful progressively enlarging right parietal scalp mass	Skull radiography and CT demonstrated an expansile right parietal calvarial lesion with radiating trabecular architecture consistent with intraosseous hemangioma. Brain MRI showed a contrast‐enhancing calvarial lesion with diploic expansion. SWI revealed multiple punctate supratentorial lesions and bilateral cerebellar lesions compatible with CCMs. Spinal MRI demonstrated a cervical intramedullary cavernous malformation.	Seizure‐free for 6 months on levetiracetam; persistent intermittent headache; neurologically intact
Clinically asymptomatic sibling	16/M	Heterozygous NM_004912.4: c.2dup, p.(Met1IlefsTer31)	Clinically asymptomatic	Brain MRI demonstrated multiple CCMs with multifocal susceptibility lesions. Ophthalmological examination normal	Asymptomatic carrier under radiological surveillance
Mother	Adult/F	Variant not detected	Asymptomatic	Neuroimaging not performed	Clinically unaffected, non‐carrier
Father^a^	Deceased (41 y)/M	Not available	Fatal intracerebral hemorrhage	Imaging unavailable	Deceased; suspected affected family member

*Note:* Variant nomenclature is based on the GenBank reference transcript NM_004912.4.

Abbreviations: CCM, cerebral cavernous malformation; CT, computed tomography; F, female; M, male; MRI, magnetic resonance imaging; SWI, susceptibility‐weighted imaging.

^a^ Genetic testing could not be performed in the father because he was deceased.

### 3.2 Molecular Findings

WES identified a heterozygous frameshift duplication variant in *KRIT1*, NM_004912.4: c.2dup (GRCh37/hg19: chr7:91871448dupA), predicted to result in an early truncating protein change, p.(Met1IlefsTer31). The variant affects the translation initiation region and is expected to cause loss of normal protein function through an early loss‐of‐function mechanism. Variant interpretation using Franklin/Genoox supported a likely pathogenic classification according to ACMG criteria based on predicted loss of function in a gene where loss‐of‐function is an established disease mechanism (PVS1), absence from population databases including gnomAD (PM2), a phenotype highly specific for familial cerebral cavernous malformation (PP4), and supportive segregation evidence (PP1). No pathogenic or likely pathogenic variants were identified in the other established CCM‐associated genes, including CCM2 and PDCD10.

**FIGURE 3:**
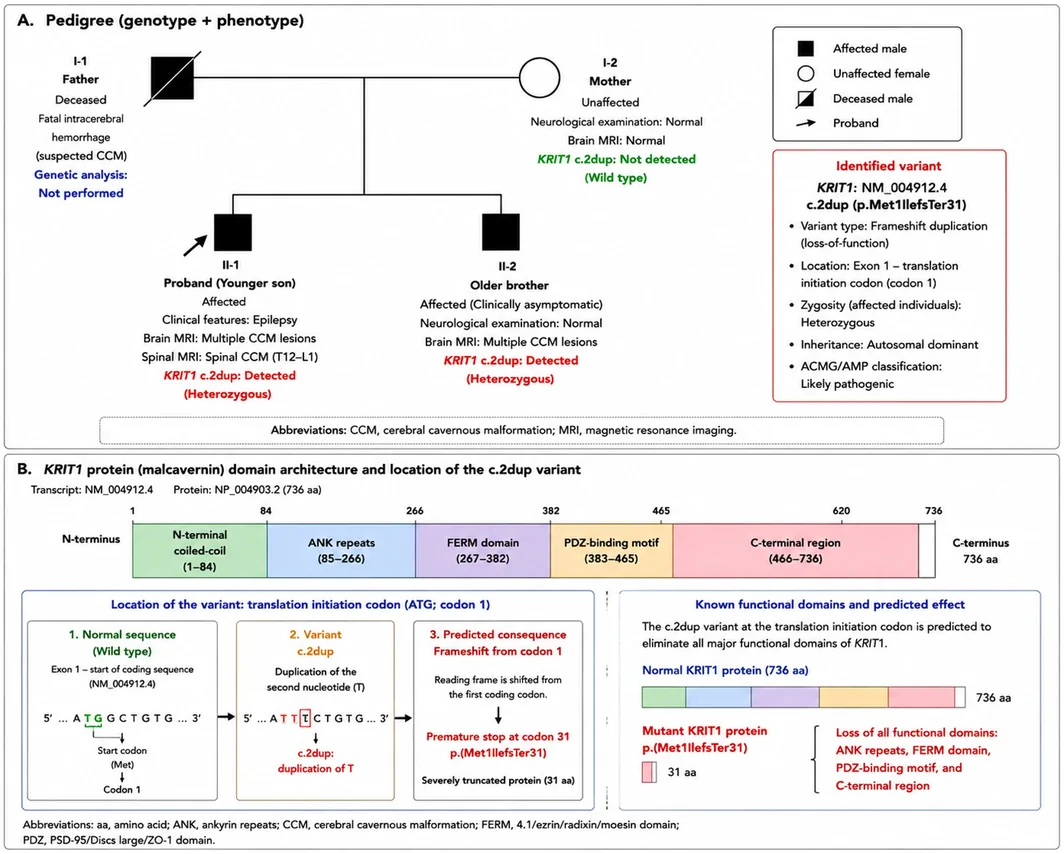
Genotype–phenotype correlation and predicted pathogenic mechanism of the novel KRIT1 c.2dup (p.Met1IlefsTer31) variant. (A) Pedigree of the reported family illustrating the segregation of the novel KRIT1 c.2dup (p.Met1IlefsTer31) variant together with the corresponding clinical phenotype. The proband and his clinically asymptomatic brother both carry the heterozygous KRIT1 variant and demonstrate cerebral cavernous malformations on brain MRI, whereas the unaffected mother does not carry the variant. The deceased father had a clinical history highly suggestive of familial cerebral cavernous malformation but was unavailable for genetic testing. (B) Schematic representation of the KRIT1 (malcavernin) protein domain architecture and the predicted molecular consequence of the identified variant. The c.2dup variant affects the canonical translation initiation codon (ATG; codon 1), resulting in a frameshift from the first coding codon and a premature termination codon at amino acid 31 (p.Met1IlefsTer31). The predicted truncated protein lacks all major functional domains, including the ankyrin repeat (ANK), FERM, PDZ‐binding motif, and C‐terminal regions, supporting a loss‐of‐function mechanism consistent with KRIT1‐associated familial cerebral cavernous malformation. Aa, amino acid; ANK, ankyrin repeats; CCM, cerebral cavernous malformation; FERM, 4.1/ezrin/radixin/moesin domain; NMD, nonsense‐mediated mRNA decay; PDZ, PSD‐95/Dlg/ZO‐1. Variant nomenclature is based on the GenBank reference transcript NM_004912.4.

Segregation analysis demonstrated the same variant in the clinically asymptomatic brother, who had MRI‐confirmed multiple cerebral cavernous malformations, whereas the mother did not carry the variant (Figure 3 and Figure S1). The father's genotype could not be evaluated because he was deceased. A structured literature search using PubMed, ClinVar, and HGMD databases did not identify previously published individuals or families specifically reported to carry *KRIT1* c.2dup or closely related translation‐initiation region frameshift variants.

The domain architecture of the *KRIT1* protein and the predicted functional consequences of the identified variant are illustrated in Figure 3.

## 4 Discussion

Familial CCM is an autosomal dominant vascular disorder characterized by incomplete penetrance and marked intrafamilial phenotypic variability (Zhang et al. 2020; Ricci, Cerase, et al. 2021; Haasdijk et al. 2012). In the present study, we identified a previously unreported *KRIT1* initiation‐region duplication variant, NM_004912.4: c.2dup, in a family with clinically and radiologically confirmed familial CCM. According to ACMG/AMP criteria, the variant fulfilled PVS1, PM2, PP4, and PP1 supporting evidence codes, consistent with a likely pathogenic classification. The variant affects the translation initiation region of *KRIT1*, a gene in which loss of function is the established disease mechanism underlying CCM. The variant segregated with disease in both affected siblings, was absent in the unaffected mother, and was not identified in population databases. Furthermore, the observed phenotype was highly consistent with familial CCM. Together, these findings support classification of *KRIT1* c.2dup as a likely pathogenic variant and expand the mutational spectrum associated with *KRIT1*‐related CCM.

Pathogenic *KRIT1* variants typically result in haploinsufficiency through a loss‐of‐function mechanism that disrupts the *Rap1–KRIT1–CCM2* signaling complex, leading to impaired endothelial junction stability, altered vascular permeability, and cavernoma formation (Yang et al. 2016; Lucas et al. 2003; Baranoski et al. 2016; Marchuk et al. 2003; Zhang et al. 2020; Chen et al. 2023; Pileggi et al. 2010; Denier et al. 2004; Stockton et al. 2010). The identified c.2dup variant introduces a frameshift at the translation initiation codon and is predicted to result in p.(Met1IlefsTer31), causing loss of all major downstream functional domains. Although functional validation studies were not performed, the predicted molecular consequence is consistent with the established pathogenic mechanism of *KRIT1*‐associated CCM.

To further support the pathogenic interpretation of the identified variant, we compared our findings with previously reported translation‐initiation and early truncating *KRIT1* variants associated with familial cerebral cavernous malformation (Table 2) (Sahoo et al. 2001; Cavé‐Riant et al. 2002; Gianfrancesco et al. 2007; D'Angelo et al. 2011; Cheng et al. 2021). Consistent with previous reports, these variants are predicted to result in loss of function through disruption of translation initiation, premature truncation, or nonsense‐mediated mRNA decay. Among reported translation‐initiation and early truncating variants identified in our literature review, the present *KRIT1* c.2dup variant is distinguished by simultaneously disrupting the canonical translation initiation codon and introducing an immediate frameshift, leading to a premature termination codon at amino acid 31 and predicted complete loss of functional *KRIT1* protein.

**TABLE 2:** Only previously reported *KRIT1* variants sharing a molecular mechanism similar to the present variant (translation‐initiation disruption or early truncating loss‐of‐function) are included.

Variant (HGVS cDNA)	Protein change	Variant type	Predicted consequence	Clinical phenotype
c.1A >G	p.(Met1Val)/p.0?	Start‐loss	Loss of translation initiation	Familial CCM
c.143dup	p.(Arg49GlufsTer15)	Frameshift	Early truncation (predicted NMD)	Familial CCM
c.152_155del	p.(Lys51IlefsTer13)	Frameshift	Early truncation (predicted NMD)	Familial CCM
c.196C >T	p.(Gln66Ter)	Nonsense	Premature stop codon (predicted NMD)	Familial CCM
c.268C >T	p.(Arg90Ter)	Nonsense	Premature stop codon (predicted NMD)	Familial CCM
c.2dup (Present study)	p.(Met1IlefsTer31)	Translation‐initiation frameshift	Disruption of the initiation codon with immediate frameshift and predicted nonsense‐mediated mRNA decay	Familial CCM

Abbreviations: CCM, cerebral cavernous malformation; NMD, nonsense‐mediated mRNA decay.

Collectively, these observations further support haploinsufficiency as the predominant disease mechanism underlying *KRIT1*‐associated familial CCM.

Previous studies have demonstrated that susceptibility‐sensitive MRI sequences may detect clinically silent lesions in asymptomatic relatives (Akers et al. 2017; Geraldo et al. 2023; Sahin et al. 2015). In our family, SWI revealed a greater lesion burden than was apparent on conventional MRI sequences in both affected siblings, further supporting the familial nature of the disease (Akers et al. 2017; Nikoubashman et al. 2015). This observation highlights the importance of structured radiological surveillance even in asymptomatic variant carriers (Geraldo et al. 2023; Nikoubashman et al. 2015). Our findings support the incorporation of genetic testing and MRI‐based surveillance into the routine evaluation of at‐risk relatives, as early identification of affected individuals may facilitate appropriate counseling, risk assessment, and long‐term monitoring.

A notable feature of this family was the marked phenotypic variability observed among individuals carrying the same *KRIT1* variant. Clinical manifestations ranged from asymptomatic radiological disease in one sibling to focal epilepsy in the proband and a family history of fatal intracerebral hemorrhage in the father, although his genotype could not be determined. Similar variability has been reported in familial CCM and highlights the limited ability of genotype alone to predict clinical severity, hemorrhagic risk, or long‐term outcome (Lucas et al. 2003; Ricci, Cerase, et al. 2021; Denier et al. 2004).

The clinical management of familial CCM remains largely phenotype‐driven and should be individualized according to symptom burden, lesion location, hemorrhagic history, and radiological progression. In the present family, the proband's epilepsy was successfully controlled with antiseizure medication, whereas the asymptomatic sibling required only clinical and radiological surveillance. This management strategy reflects current recommendations, which generally favor conservative follow‐up for neurologically stable patients and reserve surgical intervention for selected patients with recurrent hemorrhage, progressive neurological deficits, medically refractory epilepsy, or surgically accessible symptomatic lesions (Akers et al. 2017; Alexiou and Prodromou 2011). Importantly, the substantial phenotypic heterogeneity observed within this family highlights that treatment decisions should be guided by clinical presentation rather than genotype alone.

Another noteworthy finding in our patient was the coexistence of multiple CCMs and a calvarial intraosseous hemangioma. However, current evidence does not support a causal association between *KRIT1* pathogenic variants and hemangioma formation. According to the International Society for the Study of Vascular Anomalies (ISSVA) classification, hemangiomas and cavernous malformations represent distinct vascular entities; hemangiomas are vascular tumors characterized by endothelial proliferation, whereas cavernous malformations are slow‐flow vascular malformations resulting from abnormalities in vascular morphogenesis (Willis and Sander 2016). Consistent with this distinction, large genetically characterized CCM cohorts have not demonstrated a reproducible association between CCM‐related genes and hemangiomas. In a recent study, Benichi et al. (2025). evaluated 257 pediatric patients harboring 786 cerebral and 14 spinal cavernous malformations and did not report concomitant hemangiomas or other extra‐neurological vascular tumors. Therefore, the coexistence of a calvarial intraosseous hemangioma and familial CCM in our patient is currently best interpreted as an incidental finding rather than a manifestation of the underlying *KRIT1* variant. Although this finding is most likely incidental, documenting such associations may contribute to future efforts aimed at refining the phenotypic spectrum of *KRIT1*‐related disease.

This study has several limitations that should be acknowledged. First, although the family history strongly supports an inherited form of cerebral cavernous malformation, molecular genetic data from the deceased father were unavailable, precluding complete segregation analysis within the family. Second, confirmatory Sanger sequencing was not performed. However, the identified *KRIT1* variant was detected by high‐quality WES with excellent sequencing metrics and demonstrated complete segregation with the disease phenotype among all available family members, supporting the reliability of the molecular findings. Third, the duration of clinical and radiological follow‐up was relatively short, limiting assessment of long‐term disease progression, hemorrhagic risk, and age‐dependent phenotypic manifestations. Finally, functional validation studies, including transcript and protein expression analyses (e.g., qRT‐PCR and protein‐based assays), were not performed because RNA and protein samples were unavailable from this retrospective cohort. Therefore, the pathogenicity of the identified *KRIT1* variant was inferred from its predicted loss‐of‐function effect, high‐quality WES data, familial segregation, characteristic neuroimaging findings, and ACMG/AMP‐based variant interpretation rather than direct experimental evidence. Future studies incorporating transcript, protein, and functional analyses will be important to further clarify the biological and clinical significance of this novel variant.

## 5 Conclusion

This report highlights the value of integrating molecular diagnostics, cascade family screening, and susceptibility‐sensitive neuroimaging in the evaluation of pediatric familial CCM. The identification of the previously unreported *KRIT1* c.2dup variant expands the mutational spectrum of *KRIT1*‐associated familial CCM. The marked intrafamilial variability observed in this family illustrates the limitations of predicting clinical severity solely by genotype. From a clinical perspective, these findings support early molecular diagnosis, cascade genetic screening, and structured MRI surveillance, particularly using susceptibility‐weighted imaging for sensitive lesion detection. Management should remain individualized according to lesion location, hemorrhagic history, and evolving clinical symptoms.

## Author Contributions

**Özlem Yayıcı Köken:** conceptualization, investigation, project administration, data curation, supervision, writing – review and editing, writing – original draft. **Mehpare Sarı Yanartaş:** investigation, data curation, writing – review and editing. **Hande Aygün:** investigation, writing – review and editing, data curation. **Ahmet Cevdet Ceylan:** methodology, investigation, formal analysis, validation, writing – review and editing, resources. **Kamil Karaali:** investigation, visualization, writing – review and editing. **Mehmet Saim Kazan:** investigation, resources, writing – review and editing.

## Funding

The authors have nothing to report.

## Disclosure

Reporting Guidelines: This clinical report was prepared with reference to the CARE reporting guidelines where applicable.

## Ethics Statement

Ethical approval was not required for this single‐family clinical report under institutional policy. The study was conducted in accordance with the Declaration of Helsinki.

## Consent

Written informed consent was obtained for publication of clinical, radiological, histopathological, and genetic data, including clinical images and family‐based genetic findings.

## Conflicts of Interest

The authors declare no conflicts of interest.

## Supporting information


**Figure S1:** Integrative Genomics Viewer (IGV) visualization and segregation analysis of the *KRIT1* NM_004912.4: c.2dup variant. (a) Proband demonstrating the heterozygous *KRIT1* c.2dup variant affecting the translation initiation region. (b) Clinically asymptomatic sibling carrying the same heterozygous *KRIT1* c.2dup variant. (c) Mother showing only the reference allele without evidence of the variant. Read‐level visualization supports heterozygosity in both affected siblings and absence of the variant in the unaffected mother, consistent with familial segregation of the *KRIT1* variant.

## Data Availability

The data supporting the findings of this study are available from the corresponding author upon reasonable request. Genetic and clinical data are not publicly available due to privacy and confidentiality considerations. The corresponding author had full access to all data included in this study and takes responsibility for the integrity of the data and the accuracy of the data analysis. Submission of the identified *KRIT1* variant, NM_004912.4: c.2dup, to ClinVar is currently in progress. The accession number will be provided to the journal as soon as it becomes available.
